# *Helicobacter pylori* Outer Membrane Vesicles and Extracellular Vesicles from *Helicobacter pylori*-Infected Cells in Gastric Disease Development

**DOI:** 10.3390/ijms22094823

**Published:** 2021-05-01

**Authors:** María Fernanda González, Paula Díaz, Alejandra Sandoval-Bórquez, Daniela Herrera, Andrew F. G. Quest

**Affiliations:** 1 Center for studies on Exercise, Metabolism and Cancer (CEMC), Laboratory of Cellular Communication, Program of Cell and Molecular Biology, Faculty of Medicine, Institute of Biomedical Sciences (ICBM), Universidad de Chile, Santiago 8380453, Chile; mfe.gonzalez@gmail.com (M.F.G.); pdiaztm@gmail.com (P.D.); alesandoval@gmail.com (A.S.-B.); dfhr23@gmail.com (D.H.); 2 Advanced Center for Chronic Diseases (ACCDiS), Faculty of Medicine, Universidad de Chile, Santiago 8380453, Chile; 3 Corporación Centro de Estudios Científicos de las Enfermedades Crónicas (CECEC), Santiago 7680201, Chile

**Keywords:** *Helicobacter pylori*, extracellular vesicles, outer membrane vesicles, virulence factors, inflammation, gastric cancer

## Abstract

Extracellular vesicles (EVs) are cell-derived vesicles important in intercellular communication that play an essential role in host-pathogen interactions, spreading pathogen-derived as well as host-derived molecules during infection. Pathogens can induce changes in the composition of EVs derived from the infected cells and use them to manipulate their microenvironment and, for instance, modulate innate and adaptive inflammatory immune responses, both in a stimulatory or suppressive manner. Gastric cancer is one of the leading causes of cancer-related deaths worldwide and infection with *Helicobacter pylori* (*H. pylori*) is considered the main risk factor for developing this disease, which is characterized by a strong inflammatory component. EVs released by host cells infected with *H. pylori* contribute significantly to inflammation, and in doing so promote the development of disease. Additionally, *H. pylori* liberates vesicles, called outer membrane vesicles (*H. pylori*-OMVs), which contribute to atrophia and cell transformation in the gastric epithelium. In this review, the participation of both EVs from cells infected with *H. pylori* and *H. pylori*-OMVs associated with the development of gastric cancer will be discussed. By deciphering which functions of these external vesicles during *H. pylori* infection benefit the host or the pathogen, novel treatment strategies may become available to prevent disease.

## 1. *Helicobacter pylori*, Inflammation and Gastric Cancer

*Helicobacter pylori* (*H. pylori)* is a Gram-negative, microaerophilic, spiral bacterium, which colonizes the epithelium of the human stomach [1]. *H. pylori* infection is one of the most common infectious diseases, affecting approximately 50% of the world’s population [2]. Infection rates differ according to geographic region, remaining higher in developing countries (>85%) [3]. The prevalence of *H. pylori* infection is associated with low socioeconomic status and poor sanitary conditions, which are considered risk factors [4]. The most likely route of transmission is oral-fecal, especially through contaminated food [5]. In the last three decades, the incidence of *H. pylori* infection has remained constant or even increased due to population growth, re-infection and increasing antibiotic resistance [6].

*H. pylori* was described for the first time by Warren and Marshall in biopsies from patients with gastritis and peptic ulceration [7]. Numerous studies have identified *H. pylori* as the principal etiologic agent associated with the development of chronic gastritis, peptic ulcer disease, mucosa-associated lymphoid tissue lymphoma and gastric cancer [8]. Recently, *H. pylori* has also been associated with diseases outside the gastrointestinal tract, such as, immune thrombocytopenic-purpura, iron-deficiency anemia, vitamin B12 deficiency, neurodegenerative disorders and metabolic syndrome [9].

*H. pylori* colonization of the human stomach and its role as a bacterial carcinogen is a complex process involving communication between host cells, the microorganism and the host environment [10]. In the initial phase of infection, *H. pylori* neutralizes the acidic environment of the stomach by liberating urease, which hydrolyzes urea to generate carbon dioxide and ammonia [11] that neutralize the acidic microenvironment surrounding the bacteria [12]. In addition, ammonia produces various alterations in host cells by affecting vesicular membrane transport, protein synthesis and ATP production, among others [11]. Moreover, urease activity regulates *H. pylori*-macrophage interactions, by modulating the phagosome pH and megasome formation, which are necessary for *H. pylori* survival [12].

*H pylori* has 4–8 flagella and chemotaxis receptors which it uses to colonize the stomach lining and reach epithelial cells [13]. Colonization by *H. pylori* leads to inflammation and neutrophil infiltration [13,14]. The flagellar filament of *H. pylori* is mainly composed of the structural proteins FlaA, and FlaB [12]. These flagellins are the first targets of antibodies generated by the host adaptive immune response [13]. However, flagellins, particularly FlaA, are not recognized by toll-like receptor 5 (TLR5) [15,16]. This ability of *H. pylori* to evade the immune system, contributes to the persistence of the bacteria in the gastric epithelium [13].

*H. pylori* attaches to the gastric epithelium by producing adhesins, which interact with host cell receptors [12]. These specific interactions protect the bacteria from displacement by forces produced during peristalsis, leading to successful and persistent infection [11,12]. After colonization, virulence factors, such as cytotoxin-associated gene A (CagA) and vacuolating cytotoxin A (VacA), cause further damage to the host epithelium and may increase the risk of gastric disease [10]. The cag pathogenicity island (cagPAI) is a region of the bacterial chromosome, coding CagA and the Cag type IV secretion system (T4SS), which are detectable in more virulent *H. pylori* strains [10]. Accordingly, the presence of cagPAI has been associated with higher levels of inflammation in the stomach [11].

CagA is an oncoprotein produced in the cytoplasm of the bacteria, which is then injected into host cells via the T4SS [12,17]. Once in the cytoplasm, CagA localizes to the inner surface of the plasma membrane where it is phosphorylated on tyrosine residues by c-SRC and c-ABL kinases [10]. CagA then can modify host cell signaling in multiple ways, such as by activating PI3K/AKT [18], Wnt/β-catenin [19] and MAPK/ERK signaling [10]. CagA also modulates cell adhesion and migration by binding to the phosphatase SHP-2 [12,20]. Other effects include the alteration of epithelial cell polarity and the dysregulation of apical-junctional complexes [10,21]. In addition, CagA activates NF-κB-dependent inflammatory signaling and interleukin-8 (IL-8) production in infected gastric cells [10,22]. This leads to recruitment of neutrophils and macrophages, which further increases the release of cytokines, as well as reactive oxygen species (ROS) and growth factors [22,23]. This microenvironment results in chronic inflammation, which promotes tumorigenesis [22].

VacA contains p33 and p55 protein subunits, which combine to generate an oligomeric complex [12] that forms selective anion channels in the host cell membrane and can release bicarbonate and organic anions into the host cytoplasm [12,24]. In this way, VacA aids *H. pylori* survival by inducing a flow of metabolic substrates from the mucosa to the stomach lumen [25]. This produces changes in the permeability of the plasma membrane and disrupts gastric epithelium cell integrity [12]. VacA also forms channels in the membranes of endosomes and mitochondria [24,26]. Moreover, VacA internalizes into cells via endocytosis, affecting endosomal maturation, among others [12,25]. In late endosomal compartments, these anion selective channels cause an increase in osmotic pressure, resulting in the formation of vacuoles [25,26,27]. In mitochondria, VacA induces changes in the mitochondrial membrane potential and release of cytochrome C leading to apoptosis [25,28]. In addition to its cytotoxic properties, VacA acts as an immunosuppressor by inhibiting the development, activation and proliferation of T cells [10,29], as well as preventing antigen presentation by MHC class II molecules in B cells [25]. Moreover, VacA delays phagocytosis, inhibiting phagosome maturation in macrophages [25,30]. All *H. pylori* strains contain genes coding for VacA; however, there are multiple alleles that confer variations in vacuolation capacity [10,31]. Studies reveal that VacA in allelic variant s1/m1 and s1/m2 strains is associated with more severe chronic inflammation than for other genotypes [25].

*H. pylori* is an established group 1 carcinogen and presence of the bacteria is therefore linked to carcinogenesis [32]. Stomach or gastric cancer is a multifactorial and heterogeneous disease, whereby gastric adenocarcinoma (glandular origin) represents the most common histological type (~90%) of all neoplasms originating in the stomach [33,34]. Other less common variants of gastric cancer include lympho-proliferative, mesenchymal and neuroendocrine tumors [33]. Gastric adenocarcinoma is preceded by progressive changes in the gastric mucosa, beginning with non-atrophic gastritis, triggered primarily by *H. pylori*, followed by multifocal atrophic gastritis, which advances to intestinal metaplasia, dysplasia, and finally invasive carcinoma [35,36]. This cascade of precancerous lesions describes a human model of intestinal-type gastric carcinogenesis, which is the major gastric adenocarcinoma subtype and represents 60% of all cases [34,37]. This intestinal-type gastric cancer is characterized by the presence of tumor cells that are arranged in irregular tubular or glandular structures [38].

The oncogenic effects of *H. pylori* in epithelial cells are attributed either directly to the toxic action of the virulence factors described above, and/or indirectly to inflammatory processes produced by the infection [39]. Gastritis caused by *H. pylori* infection is characterized by infiltration of the lamina propria with mononuclear leukocytes (chronic inflammation) and polymorphonuclear cells (acute inflammation) [36]. The immune response triggered by infection is both of the innate and adaptive type [36,39]. T helper-1 (Th1) cells and their signature cytokines, IL-1β, tumor necrosis factor (TNF)-α and particularly interferon (IFN)-γ, are essential for the development of gastritis [36,40]. Recruited neutrophils and macrophages produce large amounts of ROS and reactive nitrogen species (RNS) [39,41]. This results in an increase in oxidative stress, which causes DNA damage [39]. Loss of normal glandular tissue, or atrophy, is the result of chronic inflammation and tends to be multifocal (multifocal atrophic gastritis) [36]. The increased expression of multiple cytokines by the gastric mucosa accelerates the progression of atrophic changes in cells [39]. In advanced stages of atrophy, intestinal metaplasia occurs, where a phenotypic change in the gastric mucosa is observed [36]. Normal epithelial cells are replaced by cells with an intestinal phenotype [42]. Intestinal metaplasia is considered a condition that predisposes to malignancy [36,42]. Dysplasia is characterized by a neoplastic phenotype, both in cell morphology and architectural organization of the epithelium [36]. The progression from superficial gastritis to an atrophic, metaplastic and finally neoplastic mucosa is associated with the virulence of *H. pylori*, as well as environmental and host factors [43]. Both gastric mucosal inflammation and *H. pylori* may cause host cell genomic instability, damage of the DNA mismatch repair system, abnormal DNA methylation, dysregulation of noncoding gene expression, which all contribute to an accumulation of mutations and the loss of normal regulation of cell function [43,44].

Besides participation of the aforementioned *H. pylori* virulence factors to disease development, the bacteria also liberates outer membrane vesicles (*H. pylori*-OMVs) [45,46] that participate in such events. Biologically active *H. pylori* compounds can be introduced by *H. pylori*-OMVs into host cells where they may alter cell signaling pathways, impair cellular function, promote infection, and regulate immune responses [31,46]. In addition, *H. pylori* colonizes the epithelium of the stomach and causes pleiotropic changes in the infected cells of the stomach lining. All eukaryotic cells release host-derived extracellular vesicles (EVs) [47], and after infection by a pathogen these EVs may change in number, composition, or both. The details of these changes and, as a consequence, the biological effects such EVs may have in recipient cells are just beginning to be unraveled. As for *H. pylori* infection, little is known in this respect. However, the available evidence suggests they are important in this context. Thus, in the rest of this review we will elaborate on the contribution of *H. pylori* infected host-derived EVs and *H. pylori*-OMVs to the genesis and progression of gastric cancer.

## 2. What Are Extracellular Vesicles?

Extracellular vesicles (EVs) are membranous vesicles derived from cells that are important in local and distant intercellular communication, although initially such vesicles were thought to represent a mechanism by which cells eliminate waste products. All human cells that have been investigated are able to secrete EVs. Furthermore, this is a highly conserved process throughout evolution, from bacteria, protozoa, fungi, and plants to animals [48,49,50]. EVs were discovered in sheep reticulocytes, during their differentiation process. Johnstone’s group reported that immature sheep reticulocytes released most of their transferrin receptor in association with small membrane vesicles, through fusion of multi-vesicular bodies (MVBs) with the cell membrane [51,52]. Subsequently, it was shown that reticulocytes from other species also release EVs, which, in addition to transferrin receptors, also contain other proteins, such as acetylcholinesterase [52,53]. Although in the context of reticulocyte development it is clear why EVs were considered a means by which cells eliminated unwanted molecules, nowadays their role is viewed as being much more complex.

EVs are now widely recognized as vectors of intercellular communication that permit the exchange of many different types of molecules relevant to signaling events important during physiological and developmental, as well as pathological processes, such as infection by pathogens, inflammation or cancer [50,54,55,56,57]. EVs are highly heterogeneous but are generally segregated into two main groups: exosomes and microvesicles. Exosomes are of endosomal origin and their size varies between 30–100 nm diameter. They are generated by inward budding of the membrane of late endosomes destined to form MVBs. These intraluminal vesicles can be released to the extracellular space in the form of exosomes after fusion of the endosomal membrane with the plasma membrane [58]. Microvesicles, on the other hand, are larger in size ranging from 50–1000 nm or more in diameter. This type of vesicle is generated by outward budding and fission from the plasma membrane. However, depending on the cell of origin, these EVs are also known as ectosomes, oncosomes, migrasomes or apoptotic bodies [59,60,61].

The message conveyed by EVs is dictated by the profile of molecules that they transport within, as well as on their surface. The molecular profile of the EVs depends on the cell of origin and its physiological state. A large variety of molecules have been found in EVs, including proteins, such as growth factors, angiogenic factors, IL precursors, and nucleic acids, such as mRNA, miRNA, small interfering RNA, double stranded DNA, among others [56,57,62,63]. However, it seems that the content of RNA and short DNA is more relevant in microvesicles [64]. Furthermore, on their surface, EVs carry a variety of membrane proteins and cell surface molecules, in addition to several extracellular proteins, such as matrix-remodeling-associated protein 5, neural cell adhesion molecule L1-like protein, ADAM metallopeptidase with thrombospodin type 1 motif 6, CYR61 and tenascin C, to mention a few [65,66]. The lipid composition has also been analyzed and includes those commonly found in the plasma membrane, such as phospholipids, sphingomyelin and cholesterol [67].

Of these two groups of EVs, the biogenesis of exosomes is the most studied. There are at least two different mechanisms for the biogenesis of exosomes. The perhaps best-characterized route involves the sorting complex required for transport (ESCRT), a complex that selects and clusters ubiquitylated transmembrane proteins in microdomains via ESCRT-0 and ESCRT-I subunits, and then triggers budding and fission of this microdomain to the MVB interior via recruitment of the ESCRT-II and the ESCRT-III subunits [58,68,69]. Accessory proteins are also involved, such as ALIX and tumor susceptibility gene 101 (tsg101), among others [68]. The second mechanism is ESCRT-independent and involves specific lipids. In this case, accumulation of ceramide and the formation of microdomains in the endosomal membrane are necessary for the genesis of intraluminal vesicles. Important for this process is the activity of neutral sphingomyelinases, which hydrolyze sphingomyelin to generate ceramide that, thanks to its small polar head group, favors the deformation and bending of the endosomal membrane [70]. Furthermore, a second ESCRT-independent mechanism for endosomal EV formation has been identified, involving proteins of the tetraspanin family, such as CD9, CD63, CD81, and CD82 [68,71]. Depletion of ESCRT elements generally decreases EV release but does not prevent it completely. Similar results are obtained upon inhibition of neutral sphingomyelinase activity [69,72], demonstrating that cells generate EVs by different mechanisms. Moreover, secreted exosomes are heterogeneous because they can be derived from different MVB subpopulations that coexist in cells [73]. For example, in polarized cells, exosomes of different composition are released from the apical and basolateral sides of the cell [74]. Beyond these more generic mechanisms mentioned so far, additional mechanisms are available for the recruitment of specific proteins and lipids [75]. For instance, it has been suggested that the inclusion of cytosolic proteins into exosomes involves chaperones, such as Hsp70 and Hsc70 [76,77]. Regarding RNA content, the findings that ESCRT-II is an RNA-binding complex suggests that it may function to select RNA species that are incorporated into intraluminal vesicles [78].

Reportedly, the release of exosomes occurs in response to changes in intracellular calcium in cell lines, such as human erythroleukemia and mast cells [79,80]. Furthermore, potassium-induced depolarization appears to increase exosome release in neurons [81]. The Rab superfamily of small GTPases, known to control many intracellular pathways and the formation of membrane domains, appears also to play an important role in docking and tethering during exosome biogenesis. Specifically, the silencing of Rab27a and Rab27b reduces exosome release and docking of MVBs to the plasma membrane. In addition, when Rab27a is silenced, the size of MVBs increases. Moreover, when Rab27b expression is reduced, MVBs are redistributed toward the perinuclear region. In addition, silencing of the Rab27 effectors Slp4 and Slac2b, reduced exosome release [82]. These results indicate that both Rab27a and Rab27b are important for exosome release but have different specific functions. As to the regulation of MVB fusion with the plasma membrane, less is known. This process is likely to be facilitated by SNARE proteins [58].

After exosomes are released, their function depends on the interaction with the target cell. Exosomes contain adhesion proteins, including integrins, that facilitate exosome adhesion to recipient cells. In this manner, exosomes can induce changes through direct contact with the cell surface, as well as by fusing with the plasma membrane, resulting in the release of their contents into the cytosol. Additionally, upon surface binding exosomes may enter cells through internalization by endocytosis or phagocytosis. The type of interaction appears to be influenced by the origin of the exosomes and the nature of the recipient cell. When exosomes are internalized, they can be degraded, which will lead to the recycling of their content to fuel the metabolism of the recipient cell. Alternatively, they can fuse with the endosomal membrane of the recipient cell and thereby release their content into the cytosol to modulate cell function [58].

A variety of functions have been attributed to EVs. Exosomes released by cardiomyocytes under hypoxic conditions are enriched with angiogenic and pro-survival factors. Cardiac stem cells release exosomes with cardioprotective and regenerative properties, being potentially useful in cell-free cardiotherapy [83]. In cancer cell lines, inhibition of microvesicle release decreases the appearance of metastasis [84,85]. There is also evidence showing that exosomes secreted by tumors contribute to tumor progression by decreasing immune responses, promoting angiogenesis, inducing proliferation of cells, promoting extracellular matrix remodeling, or increasing migration, which favors metastasis [69,86,87]. For a more detailed overview of this topic, please consider the following literature [70,88,89,90].

## 3. Extracellular Vesicles in Host–Bacteria Interactions

During infection, EVs are released from pathogen-infected host cells (EVs) and also shed directly from pathogens, in which case they are referred to as outer membrane vesicles (OMVs). Both, host-derived EVs and pathogen-derived OMVs contribute to the dissemination of pathogenic components (e.g., pathogen-associated molecular patterns (PAMPs) including, proteins, lipids, nucleic acids and carbohydrates) to the surrounding cells and tissues [91,92,93]. EV transport of pathogenic molecules between cells contributes to host-pathogen cross-talk, the activation and modulation of the host immune system and other cellular signaling pathways.

### 3.1. Host-Derived Extracellular Vesicles

As mentioned above, EVs (also known as host-derived EVs) play roles in cell-cell communication via the presence of specific proteins and nucleic acids, which may also serve as biomarkers of disease [94]. Moreover, EVs released by infected host cells contribute significantly to promoting an inflammatory response and therefore, to promoting the development of disease.

Much of the information available regarding cellular EV production and function during bacterial infection arises from work on *Mycobacterium sp*. Particularly, *Mycobacterium tuberculosis* PAMPs are transported from the phagosome to MVBs during macrophage cell infection. These PAMPs are also found in EVs released by infected macrophages and their content can be detected in neighboring uninfected cells [91,95,96]. In addition, exosomes may serve as antigen-presenting vesicles, which have stimulatory or tolerogenic effects [97,98]. *Mycobacterium avium*-infected macrophages release EVs that stimulate pro-inflammatory responses in uninfected macrophages [99,100]. EVs released from *Mycobacterium tuberculosis-*, *Mycobacterium bovis-* and *Mycobacterium smegmatis*-infected macrophages were also shown to be pro-inflammatory [91,101]. In particular, the *Mycobacterium* 19-kDa lipoprotein present on EVs released from *Mycobacterium tuberculosis*-infected cells was reported to drive this inflammatory response, via pattern recognition receptors, such as the TLR/MyD88 pathway [102]. In addition, EVs from *Mycobacterium bovis-* and *Mycobacterium tuberculosis*-infected macrophages can stimulate pro-inflammatory responses in vivo, as intranasal injection of mice induced TNF-α and IL-12 production, as well as recruitment of macrophages and neutrophils to the lung [91]. Macrophages treated with EVs from *Mycobacterium tuberculosis* secrete chemokines that activate naïve macrophages and induce T-cell migration in vitro [103]. These results suggest that EVs from *Mycobacterium*-infected cells can promote both recruitment and activation of immune cells in vitro and in vivo and may play a role in promoting the innate immune response upon *Mycobacterium* infection.

To the contrary, *Mycobacterium* sp. components present in EVs may also suppress immune responses. Indeed, EVs released from *Mycobacterium tuberculosis*-infected cells can partially suppress IFN-γ mediated activation of recipient naïve macrophages, a response mediated by TLR2/MyD88 signaling [104]. In addition, EVs from *Mycobacterium tuberculosis*-infected cells have also been reported to contain a member of the *Mycobacterium*-PE gene family (Rv1818c) that induced apoptosis in T cells [105]. This implies that, besides their effects in macrophages, these EVs also suppress immune responses in T cells. Available evidence also shows that EVs from *Mycobacterium tuberculosis-* or *Mycobacterium bovis*-infected cells activate antigen-specific CD4+ and CD8+ T cells in vivo, as well as promote the activation and maturation of bone marrow-derived dendritic cells [106]. These results suggest that EVs are a rich source of antigens to stimulate immune responses.

Alternatively, EVs from *Salmonella*-infected macrophages are also pro-inflammatory, by increasing TNF-α production in monocytes [91]. These EVs contain lipopolysaccharide, a known PAMP, in *Salmonella* and other Gram-negative bacteria. EVs from tumor cells infected with *Mycoplasma* induce a mixed cytokine response, including production of both IFN-α and IL-10, thereby activating inhibitory B cells [107]. Moreover, the anthrax toxin produced by *Bacillus anthracis* can be released inside EVs from an infected epithelial cell line [108]. In addition, EVs released from *Chlamydia pneumoniae*-infected cells contain tissue factor, a protein participating in blood coagulation, which has been associated with cell proliferation, migration and apoptosis [109]. Moreover, several cytotoxic and secreted proteins are associated with host vesicles released from *Chlamydia trachomatis*, which contribute to the dissemination of bacterial virulence factors [110].

### 3.2. Outer Membrane Vesicles in Host-Bacteria Interactions

Shedding of outer membrane vesicles (OMVs) was initially observed in the Gram-negative bacteria *Vibrio cholera* [111] and *Neisseria meningitides* [112]. OMVs are generated by “blebbing” of the outer membrane of the bacteria and contain proteins, membrane components as well as nucleic acids. Among other functions, the release of OMVs aids in Gram-negative bacterial survival [113,114]. Evidence has also been obtained on the shedding of OMVs by Gram-positive bacteria [115]. However, there is limited knowledge concerning the mechanisms by which OMVs interact with cell surfaces and transfer their cargos. Initially, OMVs dock at the plasma membrane of the recipient cell where they are internalized via endocytosis, or fuse with the plasma membrane [116]. OMV surface proteins interact with specific receptors, which leads to the activation of signaling cascades [58]. PAMPs are present on the surface of pathogens [117] and OMVs where they may interact with pattern-recognition receptors (PRRs) present on recipient cells [118,119,120]. This suggests that molecules on the surface of OMVs may target cells or organs in the host to create a niche for infection and survival.

OMVs from bacteria have been shown to compromise the immune defenses by inducing apoptosis of host cells. For instance, OMVs from *Neisseria gonorrhoeae* contain a porin protein (PorB), which is transported to mitochondria in host macrophages, where it triggers loss of mitochondria membrane potential and cytochrome C release, leading to caspase activation and apoptosis [121]. Moreover, cytotoxic proteins found in OMVs derived from a highly virulent *Escherichia coli* strain cause activation of caspase-9 and caspase-3 after OMV uptake by intestinal epithelial cell lines [122]. OMVs released by *Bacillus anthracis*, a Gram-positive bacterium, also contain cytotoxic proteins [123]. Particularly, the OMV-associated toxins released by this bacterium (anthrolysin and anthrax toxin polypeptide) induced toxicity in macrophages, and also in mice in vivo [123].

Additionally, bacteria-derived toxins transported by OMVs can modulate immune cell functions to promote pathogen survival. For example, cytotoxic necrotizing factor type 1 (CNF1), found in OMVs from *Escherichia coli*, reduced antimicrobial activity and chemotaxis in polymorphonuclear leukocytes [124]. In addition, the OMV-associated Cif-toxin, derived from *Pseudomonas aeruginosa*, downregulated the expression of the cystic fibrosis transmembrane conductance regulator in airway epithelial cells, thereby increasing *Pseudomonas aeruginosa* pathogenicity [125]. Additionally, *Moraxella catarrhalis* sheds OMVs that reduced TLR2-induced IL-8 production by alveolar epithelial cells [126], leading to increased bacterial survival in the lungs.

Pathogen-derived OMVs also contain different RNA classes and DNA. Depending on the type of nucleic acid and its localization in OMVs, these molecules either regulate host mRNA or activate RNA/DNA sensing receptors [127]. For instance, OMVs from *Moraxella catarrhalis* induce proliferation of tonsillar B cells and activate TLR9 via OMV-associated DNA containing CpG oligodeoxynucleotide-motifs [127]. Although some studies report on the presence of small RNAs in OMVs [128,129], only a few have provided evidence that OMV-associated RNA is responsible for modulating host cell responses. In particular, OMVs from *Pseudomonas aeruginosa* contain a specific transfer RNA-derived small RNA (tsRNA; sRNA52320), which could be detected in primary bronchial epithelial cells treated with *Pseudomonas aeruginosa* OMVs [129]. OMV-mediated transfer of sRNA52320 led to reduced lipopolysaccharide (LPS)-induced IL-8 production, providing indirect evidence that OMV-enclosed tRNAs mediated this effect. Additionally, *Pseudomonas aeruginosa*-derived OMVs significantly reduced IL-8 production in bronchoalveolar lavage fluid and lowered lung neutrophil infiltration, which was sRNA52320-dependent [129].

Besides the localized cellular effects of OMVs, at present there is growing evidence that OMVs from the host microbiota can enter the systemic circulation [130,131,132,133,134]. Particularly, the gut microbiota is highly dynamic and affected by diverse environmental factors (e.g., diet, exercise, pharmaceutical drugs). Thus, the presence of OMVs in systemic circulation outside of the gastrointestinal tract, or other microbial niches, points towards OMVs as key mediators of bacteria-host communication.

## 4. Extracellular Vesicles from *Helicobacter pylori*-Infected Host Cells. Mechanisms of Action Associated Primarily with Gastric Cancer Development

**Figure 1:**
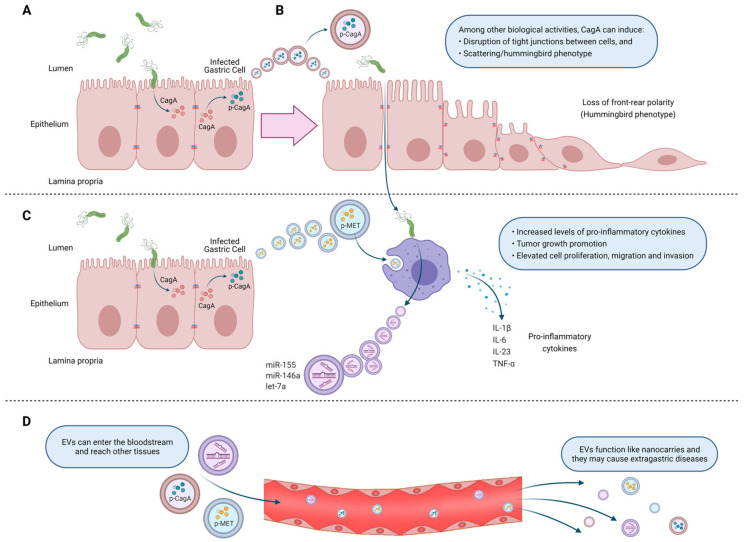
Extracellular vesicles (EVs) from *H. pylori* infected-host cells are associated with gastric cancer development. (**A**) During *H. pylori* infection, the virulence factor CagA is delivered into gastric epithelial cells. (**B**) EVs released from *H. pylori*-infected gastric epithelial cells contain CagA in its phosphorylated form (p-CagA), which induces morphological changes in host cells, causing gastric epithelial cells to elongate and spread (“hummingbird” phenotype). (**C**) Likewise, after *H. pylori* infection, EVs containing phosphorylated mesenchymal epithelial transition factor (p-MET) are released. These are internalized by macrophages, which release pro-inflammatory cytokines that promote tumor growth and increase cell proliferation, migration, and invasion. On the other hand, *H. pylori*-infected macrophages release EVs containing high levels of microRNAs, which either inhibit *H. pylori* proliferation or exert pro-inflammatory effects, depending on the microRNA type. (**D**) Finally, EVs can enter the bloodstream and reach other tissues, where they can cause extra-gastric diseases.

One of the first of the few available studies, in which exosomes were evaluated during infection by *H. pylori*, reported that CagA, one of the main virulence factors of *H. pylori*, is present in EVs isolated from the serum of infected patients (Figure 1A) [135]. Furthermore, gastric epithelial cells overexpressing CagA induced the release of EVs containing CagA in its phosphorylated form. Incubation of WT-A10 gastric epithelial cells with such EVs induced an elongated cell shape, known as the hummingbird phenotype (Figure 1B). CagA is considered an oncoprotein, which acts as a pathogenic scaffold protein, thereby altering multiple intracellular signaling pathways in infected cells. As CagA resides inside exosomes, which may transport their content to any part of the body, this mechanism can be invoked to explain how *H. pylori* triggers both local changes in the stomach and also systemic changes.

Che et al. (2018) studied exosome-mediated communication between *H. pylori*-infected gastric cancer cells and macrophages. They demonstrated that after infection with *H. pylori*, gastric cancer cells release exosomes containing phosphorylated mesenchymal epithelial transition factor (MET) (Figure 1C), which after being internalized by macrophages increased mRNA levels of the proinflammatory cytokines IL-1α and IL-6, as well as increased IL-1α secretion, which promoted tumor growth and progression in vivo. in vitro, gastric cancer cells pre-treated with the supernatant of macrophages incubated with exosomes containing MET (MET(+) exosomes) increased cell proliferation, migration and invasion, as well as the number of colonies in a clonogenic assay. Moreover, in mice injected with gastric cells treated with MET(+) exosomes, tumor volume and weight were increased compared to the tumors formed by gastric cancer cells treated with supernatants from macrophages incubated with control buffer or MET(−) exosomes. In conclusion, after infection by *H. pylori*, gastric cells secrete and transfer exosomes containing active MET to nearby immune cells, which in turn promote gastric cancer progression via IL-1α [136].

Alternatively, Chen et al., (2018) found that stimulation of gastric epithelial cells with exosomes purified from the serum of *H. pylori*-positive patients, with chronic gastritis, increased IL-1α by activating the soluble IL-6 receptor (sIL-6R). The signaling events triggered by the membrane-bound IL-6R are anti-inflammatory and protective, while to the contrary, sIL-6R signaling is proinflammatory [137]. There are only two known ways to express sIL-6R, namely either by proteolysis of the membrane-bound protein or alternative splicing [138,139]. Since in this study the authors were able to rule out the contribution of the first mechanism, they suggest that the increase in sIL-6R is mediated by alternative mRNA splicing [140].

*H. pylori*-infected macrophages, release exosomes containing elevated levels of the microRNAs; miR-155, miR-146a and let-7a (Figure 1C). To evaluate the role of microRNA-155, purified exosomes from macrophages were loaded by electroporation with microRNA-155 (Exo+miR155). Such exosomes promoted the expression of the pro-inflammatory cytokines TNF-α, IL-6 and IL-23, and also increased the expression of the proteins CD40, CD63, CD81 and MCH-I in macrophages. Additionally, treatment of macrophages with miR-155 loaded exosomes caused downregulation of MyD88 and NF-κB and inhibited intracellular *H. pylori* proliferation [141]. 

Interestingly, a metagenomic analysis of 16S rDNA sequenced in EVs isolated from gastric juice samples of patients with gastric cancer, revealed that *H. pylori* was more abundant in patients compared to the control group [142]. This finding runs contrary to previous biopsy sequencing studies, where a decrease in *H. pylori* levels was found in patients with gastric cancer [143]. Since a biopsy sample represents only a small part of the stomach while gastric juice content likely reflects events in most of the stomach, isolating EVs from gastric juice should provide a better method for diagnosing *H. pylori* than conventional biopsy-based tests. Moreover, the elevated levels of 16S rDNA from *H. pylori* identified in EVs isolated from gastric juice in patients with gastric cancer compared to healthy controls, is indicative of high levels of *H. pylori*-derived OMVs, which, as will be discussed subsequently, could promote damage to the mucosa gastric.

Beyond the more local effects in the stomach described so far, a considerable body of evidence links the presence of *H. pylori* also to the development of extra-gastric diseases, including cardiovascular diseases, such as coronary heart disease, and ischemic stroke. Furthermore, *H. pylori* presence is associated with an enhanced risk of developing diabetes mellitus, hematological diseases and Parkinson’s disease, among others (reviewed in [144]. Furthermore, epidemiological studies suggest that infection with CagA-positive *H. pylori* strains increases the risk of colorectal and pancreatic cancer [145,146].

Until recently, specific mechanisms explaining these systemic effects of *H. pylori* had not been established, although some hypotheses were put forward. For example, as a direct effect in the vascular wall, bacterial infection induces endothelial dysfunction. Alternatively, indirect effects may be attributed to increased systemic inflammation, which enhances the levels of circulating pro-inflammatory cytokines that may accelerate the development of cardiovascular diseases. In addition, recent evidence, like that reviewed here, propose a new mechanism, in which cell to cell communication mediated by EVs during *H. pylori* infection could play a central role (Figure 1D). While still rather speculative as a working hypothesis, this is certainly an area of research that merits future attention.

In summary, EV-mediated communication between *H. pylori*-infected and uninfected gastric epithelial cells, in a local context, may promote changes that favor the development and/or progression of gastric cancer. Furthermore, these EVs released by infected gastric epithelial cells modulate the responses of immune cells, like macrophages, to either promote or inhibit inflammation. Finally, these EVs can enter the bloodstream and reach other tissues, where they may cause extra-gastric diseases, since they can function as nanocarriers for molecules derived from pathogens and pathogen-infected cells.

## 5. Outer Membrane Vesicles from *Helicobacter pylori*

**Figure 2:**
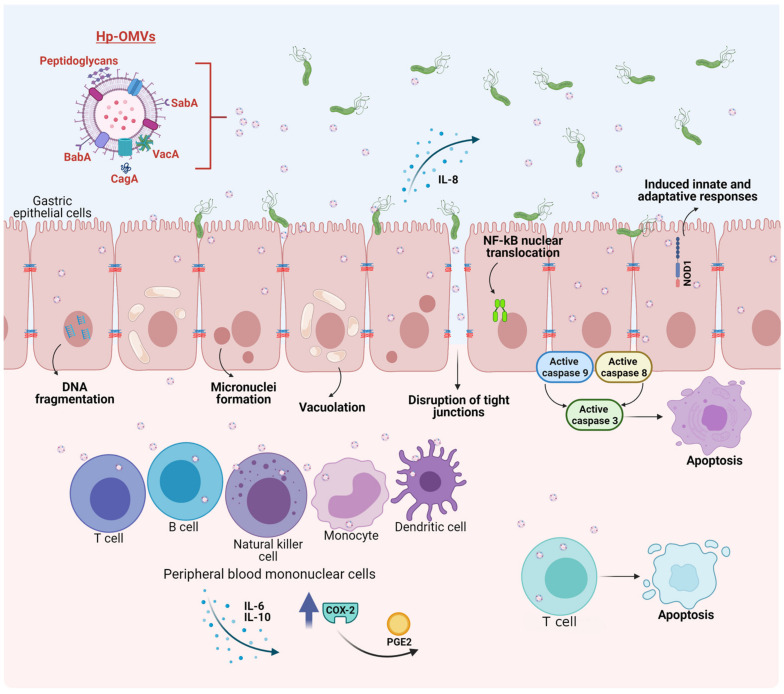
Biological effects of outer membrane vesicles released by *H. pylori* (*H. pylori*-OMVs) on host cells. SabA and BabA present in *H. pylori*-OMVs promote adherence to the gastric mucosa. Internalization of *H. pylori*-OMVs by epithelial cells leads to DNA fragmentation and activation of caspases-8, 9 and 3. *H. pylori*-OMVs induce micronuclei formation and vacuoles mediated by VacA. CagA promotes the disruption of tight junctions between cells. Moreover, *H. pylori*-OMVs induce NF-κB nuclear translocation and increase IL-8 production. Peptidoglycans from *H. pylori*-OMVs, stimulate the cytosolic nucleotide binding oligomerization domain 1 (NOD1) response. In peripheral blood mononuclear cells, *H. pylori*-OMVs stimulate proliferation and the release of IL-6 and IL-10. *H. pylori*-OMVs also induce the expression of cyclo-oxygenase-2 (COX-2) and the production of prostaglandin E2 (PGE2). Alternatively, the presence of VacA induces apoptosis in T cells.

*H. pylori*, like many of the Gram-negative bacteria mentioned above, secrete outer membrane vesicles (OMVs) derived from the bacterial outer membrane. These vesicles ranging from 20–450 nm size participate in the communication between bacteria as well as with the environment and, therefore, are important players in bacterial pathogenesis [147,148,149]. It has been reported that *H. pylori* releases OMVs both in vitro and in vivo [150,151,152,153]. OMVs secreted from *H. pylori* (*H. pylori*-OMVs) retain many of the surface molecules of the bacteria, such as LPS, peptidoglycan and outer membrane proteins [147]. Additionally, *H. pylori*-OMVs contain cytoplasmic molecules, such as proteins associated with translation and virulence factors [154]. Olofsson et al. (2010), determined that *H. pylori*-OMVs phospholipid composition is similar to that of the bacteria outer membrane and includes phosphatidylglycerol, cardiolipin, lyso-phophatidylethanolamine, phosphatidylethanolamine and cholesterol. However, some components are enriched in the OMVs compared to the bacterial outer membrane, as is the case for the protease HtrA [155]. Mass spectrometry analysis revealed that 77% of the bacterial outer membrane proteins could also be detected in *H. pylori*-OMVs. Moreover, several proteins associated with *H. pylori* virulence, such as the urease subunits, VacA, CagA, BabA and SabA adhesins were identified (Figure 2). In addition, γ-glutamyl transpeptidase, the protease HtrA, as well as the cytoplasmic proteins GroEL, catalase, metabolic and ribosomal proteins were also identified in *H. pylori*-OMVs [155].

Proteomics analysis of OMVs from *H. pylori* identified a subset of proteins that are not detected in their parent bacterium, suggesting that there is a mechanism for cargo selection [149]. In addition, the size, protein composition and cargo selection for OMVs depended on the stage of *H. pylori* growth [149]. In this study, *H. pylori*-OMVs were isolated and purified from bacterial cultures in early, late and stationary growth phase. The results show that *H. pylori*-OMVs isolated from bacteria in the late and stationary phases of growth are smaller but more abundant than *H. pylori*-OMVs from an early phase bacteria. Furthermore, OMVs isolated from *H. pylori* in early growth phase are enriched in metabolic proteins and virulence factors, such as VacA, urease and CagA compared to OMVs from later stage bacteria [149]. On the other hand, the environmental conditions and nutrient availability can also affect the composition of *H. pylori*-OMVs. When *H. pylori* was cultured on *Brucella* broth or blood agar, the derived OMVs showed a different protein pattern [155]. In addition, proteomic analysis revealed that the number of OMV associated proteins may vary between *H. pylori* strains J99 and NCTC 11637 [154]. However, the mechanisms that produce these differences are unknown.

According to epidemiological studies, there is an association between *H. pylori* infection and iron metabolism. Therefore, an unbalanced iron metabolism could affect the outcome of *H. pylori* infection [156,157,158]. Interestingly, iron availability affects the composition of *H. pylori*-OMVs. In particular, Keenan et al. (2008), determined that *H. pylori*-OMVs obtained from iron-containing media are LPS enriched, whereas *H. pylori*-OMVs from bacteria grown under iron-limiting conditions have less and shorter LPS [159]. Furthermore, iron deficiency causes a decrease in bacterial growth, but does not reduce the release of OMVs. However, OMVs released under iron deficient conditions contain less VacA, which is consistent with the absence of vacuolation observed in HEp-2 cells after incubation with these OMVs [160].

OMV formation initiates when protrusions are generated from the outer membrane of Gram-negative bacteria that then pinch off, capturing some of the periplasm content [155]. The ability of *H. pylori*-OMVs to elicit a response in the host depends on uptake and the entry into epithelial cells [148]. The internalization of OMVs secreted by *H. pylori* occurs primarily through clathrin-dependent endocytosis and, to a lesser extent, through clathrin-independent mechanisms [161,162]. Concerning cholesterol-dependent mechanisms for internalization, contradictory evidence has been obtained [161,162]. On the other hand, Parker et al. (2010) showed that within 20 min of stimulation of AGS cells with *H. pylori*-OMVs, all associated OMVs localized within the cells, and that uptake occurred at a higher rate in the presence of VacA. Additionally, the authors observed that *H. pylori*-OMV uptake is inhibited by addition of LPS [163]. More recently, Turner et al. (2018), demonstrated that the size of OMVs determines the endocytic entry mechanism. A heterogenous sized *H. pylori*-OMV population results in uptake by micropinocytosis, clathrin- and caveolin-mediated endocytosis. Generally, smaller OMVs (20–100 nm) prefer caveolin-mediated internalization, whereas larger OMVs (90–450 nm) seem to prefer clathrin- and dynamin-mediated endocytosis [148]. Interestingly, larger OMVs contain more and a wider range of proteins than smaller OMVs. For example, large OMVs, but not small OMVs contain BabA and SabA adhesins [148]. However, both populations have virulence factors, such as urease and VacA. Thus, OMV size determines the role these vesicles play in pathogenesis [148].

Accordingly, *H. pylori*-OMVs transport on their surface or inside, several virulence factors, such as VacA and CagA, and are rapidly internalized by gastric epithelial cells [93,155,163]. Additionally, both the adhesins BabA and SabA were also found on the OMV surface and shown to be biologically active as mediators of adhesion to the human gastric mucosa. There is a high variability between strains in terms of OMV protein profiles. Such variations also affect VacA presence [93,164]. VacA was identified using specific antibodies in *H. pylori*-OMVs present in gastric biopsies and *H. pylori* cultures. In addition, VacA was shown to be biologically active in OMVs, since vacuolation was observed after incubation of HEp-2 cells with OMVs from *H. pylori* 60190 (Figure 2) [151]. Additionally, VacA is internalized and can be observed intracellularly in epithelial cells when these are incubated with either the free form or bound to OMVs. Ricci et al. (2005), showed that approximately 75% of total VacA is released in the free form and the rest is in OMVs. Although both soluble VacA and OMV VacA can induce vacuolization, the OMV VacA is less effective in this respect and may perhaps play a different role [165].

Another important virulence factor identified in OMVs is urease. Proteomic analysis revealed that UreA, a urease catalytic subunit, was present in *H. pylori*-OMVs isolated from *H. pylori* strain 26695 and CCUG 17875. Moreover, in AGS cells treated with *H. pylori*-OMVs from the strain 26695, UreA localized to the cytoplasm and nucleus. Therefore, OMVs can transfer UreA to gastric cells. In addition, the nuclear targeting of UreA lead to morphological changes, suggesting that UreA has functions that are independent of its role as an enzyme [166]. Consistent with this notion, recent studies showed that urease also functions as a ligand for TLR2 to induce stabilization of hypoxia-inducible factor-1α [167].

Catalase (KatA) is a virulence factor that promotes bacterial survival by eliminating hydrogen peroxide and hypochlorite generated by immune cells [168]. Notably, catalase was identified in *H. pylori*-OMVs at a 7-fold higher concentration than in *H. pylori* itself. Thus, *H. pylori*-OMVs, are endowed with a strong anti-oxidant capacity that surrounds the bacteria and protects against oxidative damage produced by the immune system [168].

*H. pylori* like many bacteria, forms biofilms, which contain extracellular matrix components, including exo-polysaccharides, proteins, lipids and DNA. Moreover, biofilm formation is important for survival and successful infection by many pathogenic bacteria. Interestingly, OMVs are detected in the matrix of *H. pylori* biofilms and the production of *H. pylori*-OMVs was found to strongly correlate with biofilm formation [169]. Therefore, another role of OMVs may be to promote *H. pylori* biofilm formation and thereby enhance bacterial survival as well as favor infection of the stomach epithelium.

## 6. Biological Effects on Host Cells of Outer Membrane Vesicles Released by *Helicobacter pylori*. Mechanisms of Action Associated with Gastric Cancer Development

OMVs from *H. pylori* have many effects, both in vitro and in vivo [170]. In particular, *H. pylori*-OMVs have been shown to induce micronuclei formation in gastric AGS cells, indicating genomic damage, apparently mediated by VacA. In the same gastric epithelial cell line, internalization of *H. pylori*-OMVs lead to the formation of large vacuoles, as early as 4 h after incubation (Figure 2). Additionally, disruption of vacuole integrity and reduction in cellular glutathione were observed and restoring glutathione levels by the addition of glutathione ester in vitro to AGS cells prevented micronuclei formation caused by *H. pylori*-OMV stimulation, suggesting a role for oxidative stress in the infection process [153]. As mentioned previously, *H. pylori*-OMVs also contain active CagA, which appears to promote the disruption of tight junctions between epithelial cells (Figure 2), given that CagA from *H. pylori*-OMVs localizes in close proximity of zona occludens-1 protein junctions, which correlates with a disorganized and diffuse zona occludens-1 pattern after stimulation by *H. pylori*-OMVs. Moreover, CagA in *H. pylori*-OMVs enhances histone H1 affinity for ATP, which may modulate gene transcription, although this point was not addressed in the study [171].

Adhesins important for *H. pylori* adherence to the human gastric epithelium, such as SabA and BabA, are present in *H. pylori*-OMVs, and are biologically active, as they promote adherence between OMVs isolated in vitro, as well as to the human gastric mucosa in biopsies. Moreover, pre-incubation of *H. pylori*-OMVs with Leb-receptor or sLex receptor conjugates before adding OMVs to the human gastric mucosa from biopsies, prevented *H. pylori*-OMV binding, suggesting that SabA and BabA are important for adherence to the gastric mucosa [155]. The effect of *H. pylori*-OMVs on epithelial cell proliferation appears to be dose dependent. After 24 h of stimulation, low OMV doses increased AGS cell proliferation up to 30%; alternatively, at high doses, approximately 50% growth arrest and a decrease in proliferation is observed. In addition, high doses of *H. pylori*-OMVs increase toxicity and IL-8 production [163,172]. The decrease in cell viability of gastric epithelial cells after stimuli with *H. pylori*-OMVs was also corroborated by others [173]. Other effects in AGS cells observed in this study included vacuolation and apoptosis. Specifically, DNA fragmentation and activation of the caspases-8, 9 and 3 were observed but these events occurred in the absence of cytochrome C release (Figure 2) [173].

Less is known about the effects of *H. pylori*-OMVs on cells of the host immune system; however recent evidence shows that they have opposite effects. For instance, treatment with *H. pylori*-OMVs stimulate proliferation and the release of IL-6 and 10 from peripheral blood mononuclear cells [93]. Alternatively, *H. pylori*-OMVs induce apoptosis in Jurkat T cells (immortalized human T cells) and in naïve CD4+ cells [93]. *H. pylori*-OMVs also induce the expression of cyclo-oxygenase-2 in peripheral blood mononuclear cells, and as a consequence, *H. pylori*-OMVs can inhibit human T cell proliferation, via prostaglandin-E2. Induction of IL-10 was also observed in peripheral blood mononuclear cells (Figure 2) [174].

*H. pylori*-OMVs induced, in a dose-dependent manner, IL-8 in gastric AGS cells, and IL-6 as well as TNF-α in mouse macrophages. In vivo, oral and intraperitoneal administration of *H. pylori*-OMVs in mice induced IFN-α, IL-17 and IL-4 expression, as well as increased immunoglobulin G generation. In vivo, *H. pylori*-OMVs targeted stomach epithelial cells in mice. Thus, *H. pylori*-OMVs induce immune responses both in vitro and in vivo. Since, as mentioned before, *H. pylori*-OMVs are abundant in the stomach of gastric cancer patients, these can infiltrate the gastric mucosa to induce inflammation, and potentially promote gastric cancer [142].

*H. pylori*-OMVs also contain peptidoglycans, which stimulate the cytosolic nucleotide binding oligomerization domain 1 (NOD1) response in epithelial cells (Figure 2). In addition, *H. pylori*-OMVs administered intra-gastrically induced innate and adaptive immune responses in mice, via NOD1. Specifically, *H. pylori*-OMVs induce NF-κB reporter activity and nuclear translocation of the NF-κB p65 subunit, in addition to IL-8 production (Figure 2) [147]. IL-8 and IL-6 are pro-inflammatory cytokines that prolong inflammation, while IL-10 is an anti-inflammatory cytokine, which has a suppressive function on cytokine secretion. Thus, *H. pylori*-OMVs can elicit opposing effects in the immune system, by increasing or decreasing the proliferation of immune cells, as well as by elevating both, pro- and anti-inflammatory cytokines. Further research is required to determine how *H. pylori*-OMVs contribute to the development of gastric cancer associated with *H. pylori* infection.

## 7. *Helicobacter pylori*-Outer Membrane Vesicles and Extracellular Vesicles from *Helicobacter pylori* Infected-Host Cells in Vaccine Development and Biomarker Research

Recent applications of OMV and EV research include vaccine development and biomarker identification [175,176]. Currently, the first line of treatment against *H. pylori* is antibiotic combination therapy. However, in the last decades resistance to antibiotics has increased [177]. Therefore, new alternative forms of therapy are being developed, and vaccines have emerged as a potential candidate [178].

Vaccines developed against the bacterial outer membrane have been used to induce protection against various pathogens. Hence, the use of OMVs for the same purpose has recently been explored. The first challenge in developing a vaccine targeting *H. pylori*-OMVs, is the method used for isolating large quantities of OMVs. In this respect, Turner et al. (2015), described that the TolB protein is key to *H. pylori* membrane integrity and *H. pylori*-OMVs from a ΔtolB mutant strain increased the production of *H. pylori*-OMVs that were more immunogenic [179]. Keenan et al. (2000) evaluated immunogenicity of *H. pylori*-OMVs in a murine model of immune protection and such effects were attributed to increased serum immunoglobulin G antibody against lipoprotein 20 [175]. Liu et al. (2019), tested the immune protective response of *H. pylori*-OMVs obtained from the gerbil-adapted *H. pylori* strain 7.13 using a mouse model. The results showed that *H. pylori*-OMVs generate a strong humoral and mucosal immune response without inflammation that reduces *H. pylori* load, suggesting a potential use as a treatment [178]. Subsequently, the combination of *H. pylori*-OMVs with outer membrane proteins or whole cell vaccines, two commonly studied approaches against *H. pylori*, confirmed that *H. pylori*-OMVs from *H. pylori* strain 7.13 were effective as an adjuvant. The analysis demonstrated that vaccines with *H. pylori*-OMVs induced a Th2 and Th17 biased immunity, increasing cell mediated and humoral immunity, which reduces *H. pylori* colonization in a mouse model. Consequently, *H. pylori*-OMVs are effective adjuvants for these types of vaccines [180].

The high death rate from gastric cancer is due to the fact that most patients with the disease are not diagnosed until they exhibit symptoms. This makes it difficult to detect gastric cancer at an early stage [181]. Since the infection with *H. pylori* is associated with the development of gastric precancerous lesions and gastric cancer, its detection is essential for early diagnosis. Current *H. pylori* diagnosis involves both invasive and non-invasive techniques. The invasive approach requires obtaining an endoscopic biopsy, which can be analyzed enzymatically using the rapid urease test or histologically with specific antibodies or by PCR. Among the techniques for processing gastric biopsies, PCR appears to be a more sensitive method compared to the others [182]. Progress has been made in obtaining endoscopic images through the development of linked color imaging (LCI) and blue laser imaging (BLI), whereby sensitivity and specificity are on the order of 83.8–85.4% and 79.5–99.5%, respectively [183]. In addition, artificial intelligence has been used to analyze the presence of *H. pylori* in images obtained by LCI [184]. On the other hand, non-invasive tests include the urea breathing test (UBT), serology and the *H. pylori* stool antigen test. For the UBT, patients ingest labeled ^13^C-urea as a substrate for the bacterial urease [185,186]. The ^13^C-labeled carbon dioxide produced by the reaction is detected in the patient’s breath by mass spectrometry or using an isotope-selective infrared spectroscope [185]. In recent years, the development of portable mass spectrometers has reduced equipment size and the cost of the analysis [187]. Serology is useful mostly for screening and in epidemiological studies [188]. To date, none of the current *H. pylori* diagnostic options can be considered a gold standard individually. For instance, besides its invasiveness, endoscopic imaging and biopsies require specialized equipment and medical staff. Regarding the non-invasive methods, the sensitivity and specificity still needs to be improved, although values lie between 93–96% and 94–97% for the UBT and the fecal antigen test, respectively [188]. Of note, efforts have been made in the area of next generation sequencing to permit the detection of *H. pylori* mutations that confer resistance to the most commonly used antibiotics and coincide phenotypically with antibiotic susceptibility [188]. On the other hand, although it is well known that the serological test does not necessarily detect a current infection, an attempt has been made to diagnose an active infection by detecting seropositivity for 13 *H. pylori* proteins by multiplex. This study concludes that seropositivity to ≥2 of the proteins VacA, GroEl, HcpC and HP1564 is sufficient to identify an ongoing *H. pylori* infection [189]. The sensitivity limitations and the invasive nature of most diagnostic methods, together with the lack of a gold standard method, make it necessary to develop more sensitive and specific non-invasive tests for the detection of *H. pylori* infection.

EVs can potentially be used as biomarker carriers, since their content is protected from degradation in the bloodstream by their lipid membrane. These EVs can be isolated from blood or other biological fluids, such as urine, gastric juice or saliva, among others, to identify molecules associated with different diseases. EVs exhibit variations in the molecular profile in healthy subjects compared to patients with diverse diseases, and such information can be exploited for diagnosis [190]. Indeed, as mentioned before, during *H. pylori* infection, CagA is detected in EVs from patient sera [135]. However, there are not many studies that identify *H. pylori*-OMVs in circulation, mainly because it is still very difficult to differentiate pathogen-derived OMVs from EVs produced by host cells. However, some reports have revealed the presence of OMVs in circulation [130,191]. Current improvements in methods for the isolation of EVs and pathogen-derived OMVs, along with the identification of molecules that serve as biomarkers, suggest that EVs could be used as a diagnostic tool for the detection of *H. pylori* infection and preneoplastic gastric lesions in the near future.

## 8. Conclusions

During *H. pylori* infection, host cells release EVs carrying an altered profile of host-molecules, and even pathogen-derived proteins. These *H. pylori*-infected host cell EVs can have a variety of effects on gastric epithelial cells, gastric cancer cells and macrophages, including changes in epithelial cell morphology and increased release of cytokines by immune cells. In addition, during *H. pylori* infection, the bacteria releases OMVs, bearing pathogen-derived molecules, including the virulence factors CagA, VacA, Urease, LPS, SabA, BabA, among many others, that induce changes in epithelial cells, including vacuolation, micronuclei formation, disruption of tight junctions between epithelial cells, and either cell proliferation or growth arrest, depending on the OMV dose. The effects of *H. pylori*-OMVs on the immune system have been found to be contradictory, as they promote or inhibit proliferation of certain immune cells and increase pro-inflammatory and anti-inflammatory cytokine release. Overall, *H. pylori*-infected host cell EVs and *H. pylori*-OMVs can promote cellular changes that tend to favor the development and/or progression of gastric cancer. In addition, *H. pylori*-OMVs are being evaluated for use in the development of vaccines against *H. pylori* development and so far, show promising results. Finally, EVs have proven to be useful for diagnosis, as they can be isolated from various biological fluids, such as blood, urine, saliva and gastric juice, among others. Improvements in the purification of EVs and in the identification of biomarkers for detection of *H. pylori* infection, as well as preneoplastic gastric lesions, hold considerable promise for use in the diagnosis and prevention of gastric cancer. Future studies on EVs/OMVs in *H. pylori* infection should focus on the development of vaccines against *H. pylori* and early diagnosis of *H. pylori* infection along with preneoplastic gastric lesions, in order to improve the prevention of gastric cancer and the survival of gastric cancer patients.
